# Constructing Tissue-Engineered Dressing Membranes with Adipose-Derived Stem Cells and Acellular Dermal Matrix for Diabetic Wound Healing: A Comparative Study of Hypoxia- or Normoxia-Culture Modes

**DOI:** 10.1155/2022/2976185

**Published:** 2022-05-05

**Authors:** Wen Zhou, Xin Zhao, Xin Shi, Can Chen, Yanpeng Cao, Jun Liu

**Affiliations:** ^1^Clinical Nursing Teaching and Research Section, The Second Xiangya Hospital, Central South University, Changsha, China; ^2^Department of Limbs (Foot and Hand) Microsurgery, Affiliated Chenzhou Hospital, Hengyang Medical School, University of South China, Chenzhou, China; ^3^The First School of Clinical Medicine, Southern Medical University, Guangzhou, China; ^4^Department of Orthopedics, Xiangya Hospital, Central South University, Changsha, China

## Abstract

Diabetes foot ulcer (DFU) is a serious complication of diabetes, characterized by impaired vascular function, limited angiogenesis, and chronic inflammation. Direct stem cell injection on treating DFU is far from satisfactory in clinical practice, as this therapy neither protects nor localizes the injected cell suspension at the chronic ulcer site. Meanwhile, most of injected cells gradually perished within several days due to senescence or apoptosis. Acellular dermal matrix (ADM) has the potential to act as excellent cell delivery vehicles, considering it is highly biomimetic to native dermal tissue, has low immunogenicity, and suitable for stem cell attachment and proliferation. Hypoxia culture has significantly enhanced effects on the survival ability of in vitro cultured stem cells, indicating this culture mode is a suitable way for inhibiting the senescence or apoptosis of transplanted cells. In the current study, we, respectively, culture adipose-derived stem cells (ADSCs) on an ADM membrane under a hypoxia or normoxia condition to construct two kinds of tissue-engineered dressing membranes (H-ADSCs/ADM and N-ADSCs/ADM) and then comparatively evaluated their efficacy on DFU healing using a diabetic rat model. In vitro results showed that hypoxia precondition could stimulate the ADSCs secreting VEGF-A, and the culture medium from hypoxia-preconditioned ADSCs could enhance the proliferation, migration, and angiogenesis of HUVECs. In vivo results indicated that compared to the N-ADSCs/ADM membrane, the transplanted cells in the H-ADSCs/ADM membrane can survive longer at the chronic ulcer site, consequently improve angiogenesis, inhibit inflammation, and increase extracellular matrix remodeling, eventually accelerating DFU closure. This study provides an innovative covering graft for the treatment of DFU in the clinic.

## 1. Introduction

Foot ulceration is a serious complication in patient with diabetes, which affects approximately 19-34% of diabetics at least once in their life, thus poses a large burden on the patient and healthcare system [1, 2]. Regrettably, the healing process of diabetes foot ulcer (DFU) is very slow and difficult, which has a profound negative impact on the patient's quality of life [3, 4]. Currently, various therapeutic regimens are being developed, but their efficacy on DFU remains unsatisfactory [5]. Therefore, developing more effective treatment strategies is necessary.

Mesenchymal stem cells (MSCs), which having pluripotent properties and capacity for long-term self-renewal, are known to be effective in enhancing DFU healing [6, 7]. Increasing evidence has demonstrated that the therapeutic benefits of MSCs on DFU healing are mainly contributed to their ability to recruit cells; to release growth factors, chemokines, cytokines, and proteins; to promote angiogenesis; and reduce apoptosis and oxidative stress [8, 9]. According to literatures, MSCs can be isolated from peripheral blood, bone marrow, placenta, oral mucosa, amniotic fluid, umbilical cord, adipose tissue, and so on [10]. Currently, adipose-derived stem cells (ADSCs) were the most commonly used cell source in the wound healing, due to their easy accessibility, abundant sources, subcutaneous location, and longer incubation time maintaining proliferating ability and differentiation potential [11–13]. Previous studies indicated that ADSCs systemically improve angiogenesis and DFU healing via increasing epithelialization and granulation tissue formation, anti-inflammatory, and antiapoptotic effects and release of angiogenic cytokines [11, 12]. Moreover, few small clinical trials showed that ADSCs treated patients with DFU caused improved ulcer evolution, lower pain scores, and improved claudication walking distances with no reported complications [11, 12]. However, owing to the harsh environment of chronic ulcer sites, the majority of transplanted ADSCs inactivated rapidly and only a few of them are ultimately involved in the wound healing. More seriously, only part of transplanted cells can adhere to chronic ulcer site, which may hinder wound healing homogeneously and rapidly. To address these shortcomings, some researchers transplanted the MSCs into chronic ulcer site by loading them on a scaffold, and in vivo experimental results showed that this transplantation mode was effective for enhancing wound healing [14, 15]. In this transplantation mode, the scaffold acted as a three-dimensional (3D) network for cell attachment, thus ensures the transplanted cells distributed homogeneously. In addition, the scaffold provided some biochemical and mechanical signals to enhance the attached cell proliferation and differentiation, thus beneficial for DFU healing [16]. Among the scaffolds used in loading MSCs, acellular dermal matrix (ADM) has attracted wide attention owing to their low immunogenicity, high biocompatibility, good biodegradability, and high similarity to normal skin dermis in morphology and ingredients [17, 18]. Milan et.al accelerated DFU healing using ADM and human umbilical cord perivascular cells [19]. Meanwhile, Chu et.al constructed a therapeutic graft for DFU healing by seeding BMSCs on ADM [20]. In this study, we are intended to construct a tissue-engineered graft as dressing membrane for DFU healing with the mode of loading ADSCs on an ADM membrane. Regrettably, most of the transplanted BMSCs can only function about 7 days under the injured microenvironment of DFU due to cell inactivation [20]. Thus, extending the time of transplanted MSC survival in vivo may be a promising and possible solution to improve its therapeutic efficiency on DFU healing.

Oxygen concentration has been considered as a vital factor in regulating the survival, proliferation, and differentiation of MSCs [21–23]. A prior study has demonstrated that hypoxia pretreatment increases MSC survival in vivo, which would extend the function duration of transplanted cells at the wound site [22]. In addition, the conditioned media from hypoxia cultured MSCs is more effective for enhancing wound healing in comparison with normoxia-cultured MSCs because it contains more bioactive substance beneficial for cell survival and tube formation [24]. These studies imply that the hypoxia pretreatment may be an effective approach for extending the survival time of transplanted MSCs at the chronic ulcer site, thus enhancing its therapeutic efficiency on DFU healing.

In this study, two kinds of tissue-engineered dressing membranes were prepared by, respectively, culturing ADSCs on an ADM membrane under a hypoxia or normoxia condition. Additionally, a streptozotocin-induced diabetic rat model was used to comparatively evaluate the two dressing membranes on DFU healing.

## 2. Materials and Methods

### 2.1. Ethics Statement

The experimental protocol for using of Sprague-Dawley (SD) rats in our study was authorized by the Animal Ethics Committee of Chenzhou No. 1 People's Hospital (approval No. 2019045). At the end of final experiments and observations, all the rats were humanely euthanized. Fresh skin tissue used in this study was obtained free of charge with the informed consent of the donor who had been amputated in a car accident, and all procedures were approved by the Ethics Committee of Chenzhou No. 1 People's Hospital (approval No. 2022019).

### 2.2. ADSC Isolation and Identification

Inguinal subcutaneous adipose tissue was acquired from 3-week-old SD rats and washed with PBS for 3 times. After that, the adipose tissues (about 0.8 cm^3^) were minced with sterile surgical scissors and digested with 0.1% type I collagenase (Gibco, USA) for 1.5 hours at 37°C with gentle shake followed by complete medium containing 10% fetal bovine serum (FBS) to neutralize the collagenase. After the suspension was centrifuged at 1000 rpm for 5 min, the cell pellet was resuspended in complete medium (DMEM/F12, 10% FBS, 1% antibiotics, Gibco, USA) and incubated at 37°C in 5% CO_2_. Cells were passaged when reaching 80-90% confluence. The cells in passage 3 were used for further experiments.

These isolated cells were identified by flow cytometry analysis using monoclonal antibodies for CD11b, CD29, CD34, CD44, CD45, and CD73 (RAXMX-09011 Kit, Cyagen, United States). Meanwhile, these isolated cells were also identified by evaluating their potential of osteogenic, chondrogenic, and adipogenic differentiation.

### 2.3. Preparation of ADM Membrane

Fresh skin tissues were harvested from the donors at Chenzhou No. 1 People's Hospital, who were undergoing traumatic amputation owing to car accident. Additionally, we excluded in the donors with transmittable diseases, such as human immunodeficiency virus (HIV), hepatitis, syphilis, and human T-cell lymphotropic virus (HTLV).

The acquired skin tissues were incised into square shapes with 2 cm in diameter followed by PBS washing for 15 min and then decellularized within 10 mM Tris buffer containing 5 mM ethylenediaminetetraacetic acid for 12 hours. After mechanically detaching the epidermal layer from the dermis, the tissues were decellularized within 1% Triton X-100 containing 1.5 M potassium chloride and 50 mM Tris buffer for 12 hours. In order to clear the nuclear substance of the dermis, 100 *μ*g/mL RNase together with 150 IU/mL DNase was used to digest the dermis, and then, the processed dermis was rinsed in PBS for 12 hours. After the acquired ADM was sectioned into a shape of thin slice (100 *μ*m), they were lyophilized by a vacuum freeze-drier (FD8-5T, SIM, USA) and then trimmed into a circle shape with 2 cm in diameter. Finally, ADM membrane was acquired.

During decellularization, the abovementioned solutions were added with 1% penicillin-streptomycin-amphotericin B (03-033-1B, BioInd, Israel) and protease inhibitor (1 tablet/300 mL, S8820-20TAB, Sigma). Specially, protease inhibitor (1 tablet/300 mL, S8820-20TAB, Sigma) was not added into nuclease solution.

### 2.4. Evaluation of ADM Membrane

After fixed in 4% neutral paraformaldehyde for 1 day, the ADM membrane or natural dermis tissue (NDT) was embedded within paraffin and sectioned with 5 *μ*m thickness for hematoxylin & eosin (H&E), 4′,6-diamidino-2-phenylindole (DAPI), and Masson's trichrome (MT) staining. H&E staining combined with DAPI was used for observing the elimination of cellular components in the ADM membrane, while MT staining was used for observing the preservation of collagen in the ADM. In order to comparatively characterize the ultrastructural difference between ADM membrane and NDT, they were, respectively, fixed with 0.25% glutaraldehyde solution and sputter-coated with gold for scanning electron microscope (SEM) (S-3400 N; Hitachi, Japan).

### 2.5. Fabrication and Evaluation of H-ADSCs/ADM and N-ADSCs/ADM

After washing the ADM membrane (100 *μ*m thick, 2 cm diameter) within PBS for 5 min, the ADM membrane was placed on 6-well plates and a concentrated ADSC solution (1.0 × 10^6^ cells/800 *μ*L, passage 3) was seeded on the top of the ADM membrane. The plates were cultured under normoxic (21% O_2_) condition for 2 days and then refresh the culture medium. Part of plates was randomly selected and cultured under normoxic (21% O_2_) condition for 48 hours, while the other part of plates was cultured under hypoxic (1% O_2_) conditions for 48 hours. Thus, the H-ADSCs/ADM or the N-ADSCs/ADM was acquired.

After fixed in 4% neutral paraformaldehyde for 1 day, the H-ADSCs/ADM or the N-ADSCs/ADM was embedded within paraffin and sectioned with 5 *μ*m thickness for H&E and DAPI staining.

### 2.6. Collection of the Culture Medium from H-ADSCs/ADM or N-ADSCs/ADM

After harvesting the H-ADSCs/ADM or the N-ADSCs/ADM, the two kinds of culture medium were, respectively, added to a centrifugal filter unit with a 3 kDa cutoff (Millipore, USA) and centrifuged at 3000 rpm to concentrate the medium by 50-fold. After that, the concentrated hypoxia- or normoxia-culture medium was mixed with FBS-free endothelial cell medium at a ratio of 1 : 3 and marked as H medium and N medium.

### 2.7. Cytocompatibility of ADM

After 48 hours incubation under a normoxic or hypoxic condition, the live cells or the dead cells in the H-ADSCs/ADM or the N-ADSCs/ADM (*n* = 4, per group) were, respectively, stained with Calcein-AM (green) or EthD-1 (red) according to the instrument of Live/Dead Assay kit (40747ES76, Yeasen, Shanghai, China). Cell viability was calculated as follows: (live cells/total cells) × 100%. Additionally, the H-ADSCs/ADM or the N-ADSCs/ADM was washed by PBS to remove the unattached cells and then observed with SEM for the adhered cells.

### 2.8. Enzyme-Linked Immunosorbent Assay (ELISA) for Angiogenic-Related Growth Factors

The levels of vascular endothelial growth factor-A (VEGF-A), hepatocyte growth factor (HGF), and fibroblast growth factor (FGF) that were secreted by ADSCs on the ADM under normoxic or hypoxic condition were tested by ELISA. In brief, the supernatant was concentrated and measured by ELISA kits (VEGF: ab100786, Abcam, USA; HGF, ER0027, Fine Biotech; FGF, KA4283, Abnova) according to the manufacturer's instructions (*n* = 3).

### 2.9. Culture Medium from Hypoxia-Preconditioned ADSCs on HUVEC Bioactivities

The effects of culture medium from hypoxia- or normoxia-preconditioned ADSCs on the proliferation of HUVECs were tested by a CCK8 kit (70-CCK8100, MultiSciences, China) (*n* = 4). HUVECs were obtained commercially (CP-H082, Procell Life Science, China). In brief, HUVECs (1000/well) were seeded in a 96-well plate and cultured by H medium or N medium. At 0, 1, 2, and 3 days of culture, CCK8 reagent was added into the culture medium of each well and then incubated at 37°C for 2 hours. The cell proliferation was measured by absorbance at 450 nm using a microplate reader (BioTek, USA).

The effects of culture medium from hypoxia- or normoxia-preconditioned ADSCs on the migration of the HUVECs were tested using wound healing assays (*n* = 3). Briefly, HUVECs were seeded in the 24-well culture plates at 4 × 10^4^ cells per well and cultured for 48 hours in the incubator until reaching about 90% confluence. A sterile 100 *μ*L pipette tip was used to scratch a straight line. Then, H medium or N medium was added into the 24-well plate. Images were obtained at 48 hours and analyzed.

The angiogenic activity of hypoxia- or normoxia-preconditioned ADSCs was evaluated by tube formation assay (*n* = 3). HUVECs (1.5 × 10^5^/well) were seeded on a Matrigel (E1270, Sigma-Aldrich) substrate and incubated with H medium or N medium at 37°C in 5% CO_2_. After 12 hours of incubation, the cord-like structures were captured and counted by phase-contrast microscopy. HUVECs cultured without H medium or N medium were used as the control group.

### 2.10. Streptozotocin-Induced Diabetic Rat Model

Adult male SD rats (10-week-old, weighing 200-250 g) were raised in a 12-hour light/dark cycle and temperature-controlled (25°C) and humidity cage, with free access to food and water. Streptozotocin (1% STZ, 75 mg/kg, S0130, Sigma, USA) was intraperitoneally injected into the abdominal cavity of all rats and fasted for solids 18 hours, and blood glucose levels were monitored by an electronic glucometer on days 0, 3, and 7. The animals with blood glucose level higher than 15 mM, weight loss, polyuria, and polydipsia were diagnosed as diabetic and used for the creation of skin wounds. After 2-week observation, the diabetic SD rats were anesthetized with 50 mg/kg pentobarbital sodium (Sigma). After shaving the rats with an electric clipper, dorsal skin was sanitized with 5% povidone-iodine solution; then, an excisional skin wound with 2 cm in diameter was created on the dorsal by a scalpel; thus, a diabetic wound model was acquired. The diabetic wounds were covered with three dressing membranes: commercial ADM (ADM group), N-ADSCs/ADM (N-ADSCs/ADM group), and H-ADSCs/ADM (H-ADSCs/ADM group). Commercial ADM membrane was bought in Beijing Jayyalife Biological Technology Co., Ltd. (J-1, Jayyalife, Beijing, China). All operations were performed by two independent investigators (Xin Shi and Yanpeng Cao) (*n* = 8).

### 2.11. ADSC Labeling and Tracking

In order to track the implanted cells at the DFU healing site, the ADSCs in the H-ADSCs/ADM or the N-ADSCs/ADM were prestained with fluorescent solution (Vybrant™ DiO, ThermoFisher, Ireland) for 20 min at 37°C prior to implantation, respectively. At 14 days after transplantation, the rats were sacrificed to harvest the circular full-thickness skin tissue at the center of the wound, frozen sections were prepared, and the cell nucleus was stained using DAPI. A fluorescence microscope (ApoTome; Zeiss) with an excitation wavelength of 594 nm was used to detect DiO-labeled cells at the DFU healing site.

### 2.12. Diabetic Wound Closure

At days 0, 7, 14, and 21 after surgery, the wounds of each diabetic rat were photographed, and wound-size reduction was calculated by two independent investigators (Yanpeng Cao and Xin Shi) using Image-Pro Plus (version 6.0.0; Media Cybernetics Inc.). The percentage of diabetic wound closure was calculated using the equation: the percentage of diabetic wound closure = (W0 − Wt)/A0 × 100%, where “W0” indicates the initial diabetic wound area and “Wt” is the diabetic wound area at the measured timepoint (*n* = 8).

### 2.13. Histological Analysis

The diabetic rats were anesthetized 14 and 21 days after operation. The circular full-thickness skin tissue (about 2.5 cm in diameter) was incised at the center of the wound and then fixed within 4% paraformaldehyde for 24 hours. The fixed skin specimens were dehydrated in graded ethanol, cleared by xylene, infiltrated by paraffin wax and embedded within paraffin blocks. After cross-sections of 5 *μ*m in thickness were prepared from paraffin blocks, slides were stained using standard protocols for H&E and MT staining for histological analysis. H&E images were captured for wound epithelization, and MT images were used for collagen contents. For H&E images, the level of reepithelialization was calculated according to following: reepithelialization (%) = (the length of the neo − epithelium across the surface of the wound/the length of wound between wound edges) × 100%. For MT images, the level of collagen deposition in the diabetic wound was measured by calculating the mean staining intensity for collagen of wound beds by the image analysis software Image-Pro Plus 6.0 software (*n* = 8).

### 2.14. Angiogenesis Assessment

At day 7 postoperation, the diabetic rats were euthanized and skin specimens on dorsal were harvested, respectively, and the undersurface of the skin was photographed to observe the newly formed blood vessels. After that, skin specimens were fixed in 4% paraformaldehyde, immersed at 4°C in 30% sucrose overnight, and then sectioned at 20 *μ*m on a freezing microtome. The sections were blocked with PBS containing 5% of normal goat serum and 0.01% of Triton X-100 for 1 hour at room temperature. After 3 washes in PBS, samples were incubated with mouse anti-CD31 antibody (ab24590, Abcam, USA) at 4°C overnight. After that, the sections were incubated with goat anti-mouse IgG H&L secondary antibody (ab150113, Abcam, USA) at room temperature for 90 min. After staining the nuclei with DAPI, these sections were captured with a fluorescence microscope (ApoTome 2, Carl Zeiss, Germany), and vessel density was quantified from at least three random visual fields per section between the wound edges using the Image-Pro Plus 6.0 software (*n* = 8).

### 2.15. Quantitative Real-Time Polymerase Chain Reaction (qRT-PCR)

**Figure 1:**
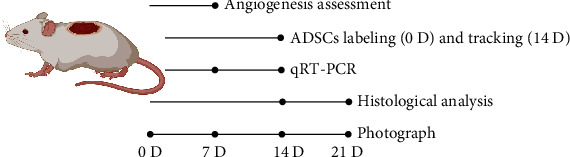
Flowchart depicting the experimental design for in vivo evaluation of ADM, N-ADSCs/ADM, and H-ADSCs/ADM in a diabetic wound rat model.

At days 7 and 14 postoperation, the diabetic rats were euthanized and skin specimens on dorsal were harvested, respectively. The total RNA of skin wound tissue was extracted using Trizol (Invitrogen, Waltham, MA, USA) according to the manufacturer's protocols. And then, cDNA was synthesized from total RNA (1 *μ*g) using Prime ScriptTM RT Master Mix (Takara Bio Inc., Shiga, Japan) at 37°C for 30 min and 85°C for 10 s. In this study, the primer sequences used for all PCR reactions are shown in Table 1, and *β*-actin was used as the housekeeping gene. The expression of the genes was normalized against the housekeeping gene *β*-actin, and the results were quantified relative to the corresponding gene expression of ADM group, which was standardized to 1 (*n* = 4).The groups, interventions, and timepoints for evaluations are listed in Figure 1.

### 2.16. Statistical Analysis

All values are represented as mean ± standard deviation. The Student *t*-test was used for the comparison between two groups, and the one-way analysis of variance with a post hoc test was used for the comparison above two groups. A value of “*P* < 0.05” was considered statistically significant. All the statistical analyses were performed using the Statistical Package for the Social Sciences 25.0 software (SPSS, United States).

## 3. Results

### 3.1. Isolation and Identification of ADSCs

**Figure 2:**
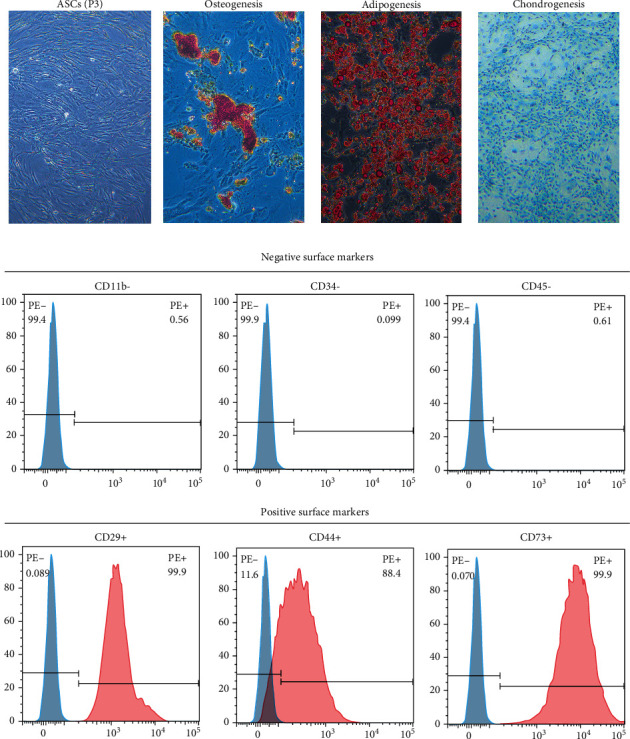
Isolation and identification of ADSCs. (a) The morphology of the isolated ADSCs from rat at passage 2. Bar = 100 *μ*m. (b) Osteogenic differentiation, chondrogenic differentiation, and adipogenic differentiation of ADSCs revealed by Alizarin Red, Alcian blue, and Oil red O staining. Bar = 100 *μ*m. (c) The expression of cell surface markers related to stem cell phenotype in ADSCs using flow cytometry analysis, which were positive for CD29, CD44, and CD73 and negative for CD11b, CD34, and CD45.

Under phase-contrast microscope, these isolated cells in the culture plate presented a spindle shape in morphology (Figure 2(a)). In addition, after osteogenic, chondrogenic, or adipogenic induction, Alizarin Red, Alcian blue, or Oil Red O images determined that the isolated cells were capable of differentiating into osteocytes, chondrocytes, or adipocytes (Figure 2(b)). Using flow cytometry analysis, we found that these isolated cells are positive for CD29, CD44, and CD73, while negative for CD11b, CD34, and CD45 (Figure 2(c)).

### 3.2. Appearance and Morphology of ADM

**Figure 3:**
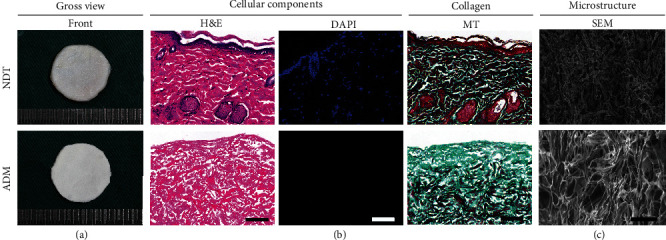
Appearance and morphology of ADM. (a) Gross appearance of acellular dermal matrix (ADM) membrane and natural dermis tissue (NDT). (b) Histological analysis of the ADM membrane and NDT. Sections were stained with H&E, DAPI, and MT. Bar = 100 *μ*m. (c) Scanning electron micrographs of the ADM membrane and NDT. Bar = 100 *μ*m.

The ADM membrane was white in color with a thickness about 100 *μ*m (Figure 3(a)). Histologically, the ADM membrane showed no cellular content in the H&E- and DAPI-stained images (Figure 3(b)). MT staining determined that the collagen content in the ADM membrane presented a similar pattern with normal dermis (Figure 3(b)). SEM images determined that the ADM well preserved the microstructure of normal dermis (Figure 3(c)).

### 3.3. Viability of ADSCs on the ADM under Normoxic or Hypoxic Condition

**Figure 4:**
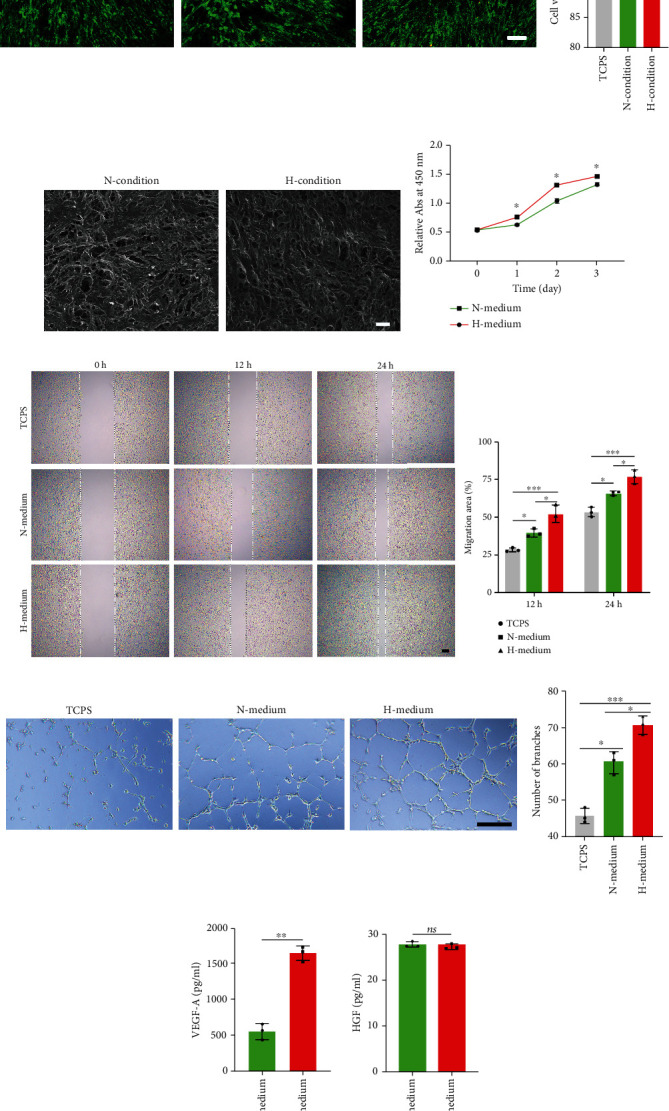
(a) Live/dead cell analysis for the ADM membrane on which ADSCs had been seeded and cultured under normoxic or hypoxic condition. Representative images show the live (green) and dead (red) ADSCs in the TCPS and the ADM membrane cultured under normoxic or hypoxic condition and the viability analysis for the cells on the TCPS and ADM membrane, *n* = 4 per group. (b) Scanning electron micrographs of the ADM membrane on which ADSCs had been cocultured under normoxic or hypoxic condition for 48 hours. (c) Proliferation of HUVECs influenced by H medium and N medium was evaluated with Cell Counting Kit-8 (CCK8) assay at 0,1, 2, and 3 days after cell seeding, *n* = 4 per group. (d) Representative images of scratch on HUVECs at 0, 12, and 24 hours after treated by H medium and N medium. The migration of HUVECs was significantly increased by H medium, *n* = 3 per group. (e) In vitro tube formation of HUVECs at 12 hours under the stimulation of C medium and H medium. Bar = 100 *μ*m, *n* = 3 per group. (f) The content of angiogenic-related growth factors (VEGF-A, HGF, and FGF) in culture medium from hypoxia- or normoxia-preconditioned ADSCs determined by ELISA, the FGF content was too low to be measured. *n* = 3 per group. All data are shown as means ± standard deviation (^∗^*P* < 0.05, ^∗∗^*P* < 0.01, and ^∗∗∗^*P* < 0.001).

Using Live/Dead assay, most of the ADSCs on the ADM under normoxic or hypoxic condition were positive for green fluorescence (live cells), while a few of ADSCs were positive for red fluorescence (dead cells). Statistically, the viability of ADSCs on the ADM membrane cultured under normoxic or hypoxic condition was lower than that on the TCPS without significant difference (Figure 4(a)). In addition, SEM images showed that a dense layer of viable ADSCs was well attached on the outer surface of ADM membrane after normoxic culture or hypoxic culture (Figure 4(b)). These results indicated that 48 hours of hypoxia culture was harmless for ADSCs on an ADM membrane.

### 3.4. Culture Medium from Hypoxia-Preconditioned ADSCs Improves the Proliferation and Migration of HUVECs

In order to evaluate the possible effect of culture medium from hypoxia-preconditioned ADSCs, CCK8 assays were performed. As showed in Figure 4(c), CCK8 assay showed that the HUVECs cultured within the H medium showed significantly higher absorption than the HUVECs cultured within the N medium at 1, 2, and 3 days (*P* < 0.05 for all). This result indicates that culture medium from hypoxia-preconditioned ADSCs could significantly stimulate the proliferation of HUVECs.

The migration-promoting effect of culture medium from hypoxia-preconditioned ADSCs was confirmed by wound healing assays. As showed in Figure 4(d), H medium and N medium both facilitated wound closure at 12 hours or 24 hours. The percentage of the migration area at 12 hours or 24 hours was significantly larger after treatment with H medium (*P* < 0.05 for all). This result implied that culture medium from hypoxia-preconditioned ADSCs could dramatically induce the migration of HUVECs.

### 3.5. Culture Medium from Hypoxia-Preconditioned ADSCs Stimulates Angiogenesis In Vitro

In order to evaluate the effect of culture medium from hypoxia-preconditioned ADSCs on angiogenic capacity, tube formation assays were performed to analyze the number of branches formed by HUVECs. As showed in Figure 4(e), the number of branches in the H medium group was significantly higher than that of N medium group (*P* < 0.05). This outcome indicates that culture medium from hypoxia-preconditioned ADSCs could facilitate angiogenesis in vitro.

### 3.6. Culture Medium from Hypoxia-Preconditioned ADSCs Contains More VEGF-A

To comparatively evaluate the content of angiogenic-related growth factors in culture medium from hypoxia- or normoxia-preconditioned ADSCs, ELISA assay was performed. As depicted in Figure 4(f), culture medium from hypoxia-preconditioned ADSCs contains more VEGF-A than culture medium from normoxia-preconditioned ADSCs (*P* < 0.05). As for HGF, no significant differences were found between the culture medium from hypoxia-preconditioned ADSCs and the culture medium from normoxia-preconditioned ADSCs. Regrettably, the FGF content was too low to be measured.

### 3.7. Morphological Appearance of H-ADSCs/ADM

**Figure 5:**
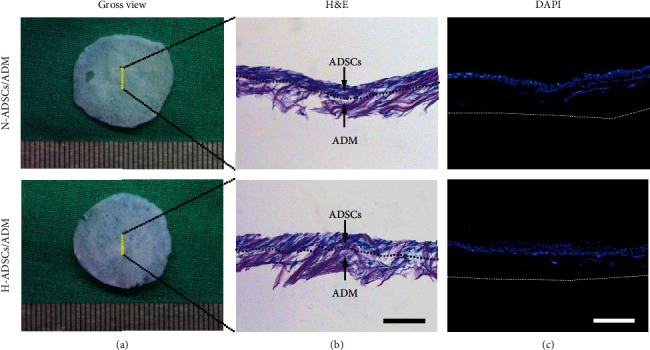
Morphological appearance of H-ADSCs/ADM. (a) Gross appearance, (b) histological morphology, and (c) DAPI images of the N-ADSCs/ADM membrane and the H-ADSCs/ADM membrane. The dotted line in (b) represents the boundary between the ADSCs and the ADM membrane. The dotted line represents the bottom of the ADM membrane. Bar = 100 mm.

Both the H-ADSCs/ADM and the N-ADSCs/ADM showed a white membranous membrane in gross appearance (Figure 5(a)). Histologically, a dense layer of ADSCs was attached on the surface of the ADM membrane with a few cells entering the deeper interstices of the ADM (Figure 5(b)). In addition, as showed in DAPI images of H-ADSCs/ADM or N-ADSCs/ADM, a dense layer of viable ADSCs was well attached on the outer surface of ADM (Figure 5(c)).

### 3.8. In Vivo Cell Tracking Analysis

**Figure 6:**
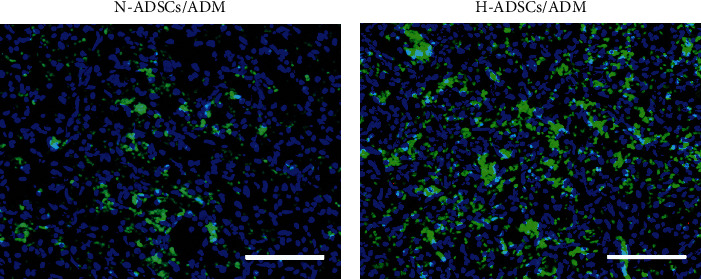
Hypoxia-precondition enhances the survival ability of ADSCs at DFU healing site. ADSC tracing in vivo at postoperative day 14. (a) Skin wound treated with N-ADSCs/ADM membrane with DiO labeling. (b) Skin wound treated with H-ADSCs/ADM membrane with DiO labeling. Bar = 100 mm.

There are more DiO-labeled ADSCs at the H-ADSCs/ADM-treated DFU healing site compared to the N-ADSCs/ADM-treated DFU healing site at postoperative day 14 (Figure 6), which implied that the hypoxia-precondition could enhance the survival ability of ADSCs at DFU healing site.

### 3.9. Wound Closure Analysis

**Figure 7:**
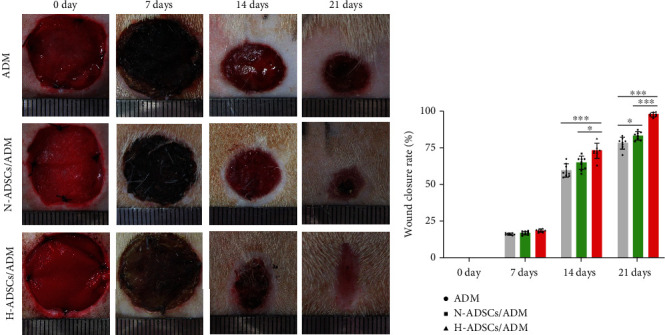
H-ADSCs/ADM facilitating diabetic wound healing. (a) Photos of the skin wound healing in diabetic rat among the ADM, N-ADSCs/ADM, and H-ADSCs/ADM groups at postoperative 0, 7, 14, and 21 days. (b) Statistical analysis of wound area among the three groups at postoperative 0, 7, 14, and 21 days, *n* = 8 per group. All data are shown as means ± standard deviation (^∗^*P* < 0.05, ^∗∗^*P* < 0.01, and ^∗∗∗^*P* < 0.001).

As showed in gross images of the wounds (Figure 7(a)), there are no apparent complications or excessive scarring in any of the conditions at any timepoint. New epidermis grew from the edge of skin wound and gradually extended to the center of skin wound, resulting in the reduction of wound area. The rats in the H-ADSCs/ADM group showed an almost complete wound closure after 21 days. The result of wound closure quantification (Figure 7(b)) showed that there are no differences among groups at days 7, while some differences were observed at day 14. Specifically, at day 21, the H-ADSCs/ADM group showed a significantly (*P* < 0.05) higher wound closure than the other groups (*P* < 0.05 for all).

### 3.10. Histological Analysis

**Figure 8:**
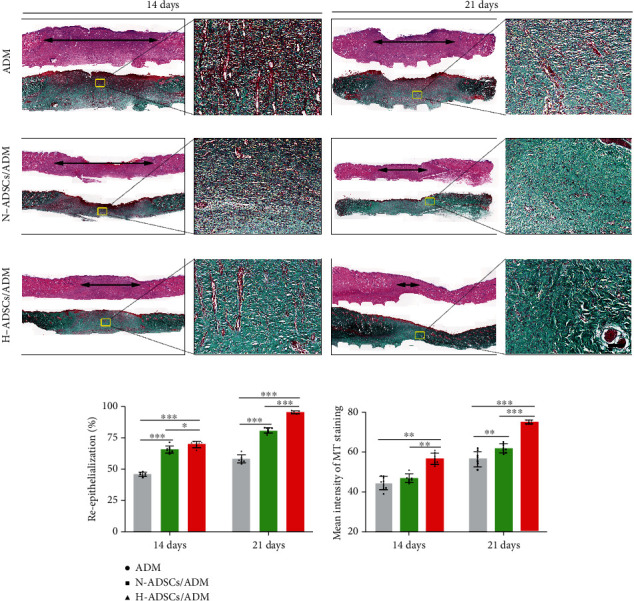
H-ADSCs/ADM facilitating reepithelialization and collagen deposition in diabetic wounds. (a) The images of H&E- and MT-stained sections in the ADM, N-ADSCs/ADM, and H-ADSCs/ADM groups at 14 or 21 days after the operation. “Double-headed black arrows” mean the edges of the scars. Bar = 100 *μ*m. (b) Quantitative analysis of the reepithelialization extent and the mean intensity of MT staining in the histological sections of the ADM, N-ADSCs/ADM, and H-ADSCs/ADM groups, *n* = 8 per group. All data are shown as means ± standard deviation (^∗^*P* < 0.05, ^∗∗^*P* < 0.01, and ^∗∗∗^*P* < 0.001).

The extent of reepithelialization of skin wounds was evaluated on the images of H&E-stained sections (Figure 8(a)). The rats in the H-ADSCs/ADM group or N-ADSCs/ADM group showed significantly higher value in the reepithelialization rate than that of the ADM group at postoperative day 14 or 21. In addition, the reepithelialization rate of the H-ADSCs/ADM group was significantly higher than the N-ADSCs/ADM group at postoperative day 21 (Figure 8(b)).

The collagen deposition and maturation of healed skin wounds were evaluated according to the images of MT-stained sections. As shown in Figure 8(a), on postoperative day 14, extensive deposition of collagen fibers could be seen in the wound bed of the H-ADSCs/ADM group or N-ADSCs/ADM group, while in the ADM group, lots of immature collagen was formed surrounding the skin fibroblasts in the ADM group. On postoperative day 21, more collagen fibers in the H-ADSCs/ADM group were deposited at the wound healing site with a pattern of thick and wavy appearance compared to the N-ADSCs/ADM and ADM groups. More importantly, the hair follicles were regenerated at the wounds of the H-ADSCs/ADM group, when compared with the N-ADSCs/ADM and ADM groups. Quantitatively, the collagen content in the H-ADSCs/ADM group was significantly higher than that of N-ADSCs/ADM and ADM groups at postoperative day 14 or 21 (Figure 8(b)). These results implied that H-ADSCs/ADM was capable of enhancing the repair of diabetic wounds.

### 3.11. H-ADSCs/ADM Membrane Stimulating Angiogenesis in Diabetic Wounds

**Figure 9:**
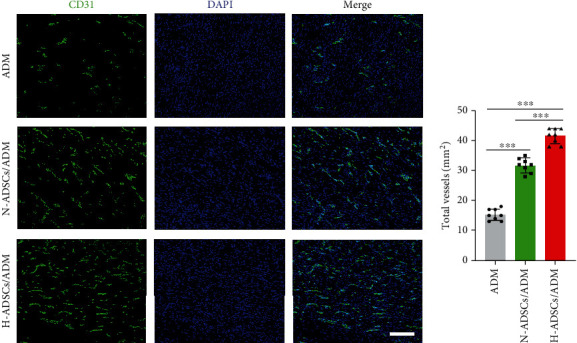
H-ADSCs/ADM membrane stimulating angiogenesis in diabetic wounds. (a) Immunofluorescence analysis of sections for CD31 showed the formation of microvessels in the ADM, N-ADSCs/ADM, and H-ADSCs/ADM groups. Bar = 100 *μ*m. (b) CD31 area quantification showed the quantitative analysis of the number of total blood vessels in wounds at day 7 postoperation, *n* = 8 per group. All data are shown as means ± standard deviation (^∗^*P* < 0.05, ^∗∗^*P* < 0.01, and ^∗∗∗^*P* < 0.001).

Angiogenesis in the DFUs was characterized by the immunofluorescence staining of CD31, a transmembrane protein expressed at the early stage of vascular development. As showed in Figure 9(a), the quantity of CD31 significantly increased in the wounds treated with the H-ADSCs/ADM membrane when compared to that of the N-ADSCs/ADM and ADM groups. The quantification analysis of CD31 showed that significantly more blood vessels were formed in wound bed of the H-ADSCs/ADM group compared to the N-ADSCs/ADM and ADM groups, and significantly more vessels were detected in the wounds of the N-ADSCs/ADM group when compared with the wounds in the ADM group (Figure 9(b)).

### 3.12. H-ADSCs/ADM Membrane Inhibiting the Gene Expression of Proinflammatory Cytokine in Diabetic Wounds

**Figure 10:**
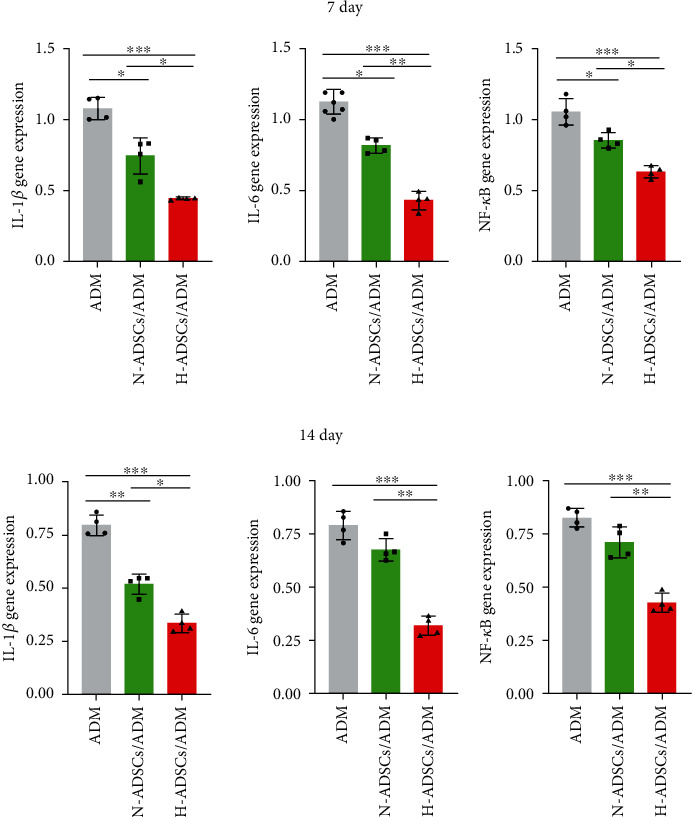
H-ADSCs/ADM membrane inhibiting the gene expression of proinflammatory cytokine in diabetic wounds. The expression of proinflammatory cytokines (IL-1*β*, IL-6, and NF-*κ*B) in the wound tissues of the ADM, N-ADSCs/ADM, and H-ADSCs/ADM groups was evaluated by qRT-PCR. The gene expression levels of IL-1*β*, IL-6, and NF-*κ*B at (a) 7 days and (b) 14 days, *n* = 4 per group. All data are shown as means ± standard deviation (^∗^*P* < 0.05, ^∗∗^*P* < 0.01, and ^∗∗∗^*P* < 0.001).

The expression of proinflammatory cytokines (IL-1*β*, IL-6, and NF-*κ*B) in the wound tissues of the ADM, N-ADSCs/ADM, and H-ADSCs/ADM groups was evaluated by qRT-PCR. Our results showed that H-ADSCs/ADM membrane significantly downregulated the expression of IL-1*β*, IL-6, and NF-*κ*B compared with the ADM and N-ADSCs/ADM groups at 7 days postwounding. Additionally, at 14 days postwounding, the expression of IL-1*β*, IL-6, and NF-*κ*B in H-ADSCs/ADM groups was obviously lower than the other two groups (Figure 10).

## 4. Discussion

DFUs are a serious complication of diabetes, which is characterized by impaired vascular function, limited angiogenesis, and chronic inflammation [25, 26]. Direct MSC injections on treating DFUs are far from satisfactory in clinical practice, as this therapy neither protects nor localizes the injected cell suspension at the chronic ulcer site [27]. Meanwhile, most of injected MSCs gradually perished within several days due to senescence or apoptosis [28]. ADM has the potential to act as excellent cell delivery vehicles, considering it is highly biomimetic to native dermal tissue, has low immunogenicity, and suitable for stem cell attachment and proliferation [29, 30]. Hypoxia culture has significantly enhanced effects on the survival ability of in vitro cultured MSCs [28], indicating this culture mode is a suitable way for inhibiting the senescence or apoptosis of transplanted cells. Therefore, in the current study, we, respectively, culture ADSCs on an ADM membrane under a hypoxia or normoxia condition to construct two kinds of tissue-engineered dressing membranes (H-ADSCs/ADM and N-ADSCs/ADM) and then comparatively evaluated their efficacy on DFU healing using a diabetic rat model. The in vivo results indicated that the transplanted cells in the H-ADSCs/ADM can survive longer at the chronic ulcer site, consequently improve angiogenesis, inhibit inflammation, and increase extracellular matrix remodeling, eventually accelerating DFU closure. This study provides a new covering graft for the treatment of DFU.

Currently, the stem cells isolated from adipose (ADSCs), bone marrow (BMSCs), or umbilical cord blood (UCB-MSCs) have seen success in accelerating diabetic wound healing. Among them, ADSCs are the most commonly used cell source, owing to its advantages of relative abundance and easy accessibility [16, 31]. As for ADSC-based strategies for wound healing, extending the survival time of transplanted cells and improving its function in stimulating angiogenesis at the chronic ulcer site are the most significant challenges. Prior studies have shown that hypoxia pretreatment can increase MSC survival and improve its function in stimulating angiogenesis [22, 28, 32]. Therefore, in the presented study, we engineered a dressing membrane by culturing ADSCs on an ADM under a hypoxia condition. In consideration that appropriate hypoxic culture conditions are of great significance for promoting MSC survival and stimulating the paracrine activities of MSCs, we firstly explored the optimal culture conditions by selecting a suitable oxygen tension. ADSCs have been shown to reside in a lower (1-5%) oxygen tension than that normally used in ambient cell culture (20-21%) [33]. Due to the lack of vascularization, in the early period of DFU healing, the oxygen tension may be lower than 4% [34]. Thus, we selected 1% O_2_ oxygen tension as hypoxia culture parameter for constructing the required dressing membrane. Stubbs et al. found that compared to normoxic culture condition, hypoxic culture condition could improve the survival of ADSCs [35]. In addition, Zhang et al. revealed that hypoxia-cultured ADSCs secreted more angiogenic factors than the normoxic cultured ADSCs [36]. Another study showed that the supernatant from hypoxia-preconditioned ADSCs was capable of improving endothelial cell survival and angiogenesis capacity [35]. Our in vitro study also showed that the supernatant from hypoxia-preconditioned ADSCs contains more VEGF-A and exhibited better function in promoting the proliferation, migration, and tube formation of HUVECs than that from ADSCs under normoxia.

Despite the difference in preparing covering grafts for the treatment of DFU, the results of the in vitro study showed that both methods had similar cell loading efficiency and viability. After cell incubation, both hypoxia culture and normoxia culture resulted in a dense layer of viable cells on the outer surface of ADM. This result indicates that both culture methods can be a harmless approach for culturing ADSCs on an ADM membrane. More importantly, the supernatant from hypoxia culture showed significantly higher bioactive molecules associated with angiogenesis compared with the supernatant from normoxia culture, indicating that hypoxia culture was an effective means for stimulating ADSCs secreting angiogenesis-related molecules.

In this study, we determined that the streptozotocin-induced diabetic rats treated with the H-ADSCs/ADM showed a significantly better healing speed in skin wound closure than the rats treated with N-ADSCs/ADM, as characterized by the improved angiogenesis, optimized local inflammation, and increased extracellular matrix remodeling. The reasons for this healing difference in skin wound may be that (1) skin wound closure is proceeded by the initial inflammatory phase, middle proliferative phase, and last remodeling phase, which involves multiple cells, growth factors, and extracellular signals [37]. In the case of diabetes, the skin wounds are usually characterized by continuous inflammation, cell dysfunction, and limited angiogenesis, resulting in delayed wound healing [16]. Owing to the ability of ADSCs differentiating into different lineages and secreting angiogenic or anti-inflammatory molecules, the potential of ADSCs for wound healing has been shown in several studies [11, 12]. This may be the important reason why the skin wound in the N-ADSCs/ADM and H-ADSCs/ADM groups closed significantly quicker than the ADM group. (2) The implanted ADSCs in the H-ADSCs/ADM could tolerate local hypoxia better than the ADSCs in the N-ADSCs/ADM and survived better at the chronic ulcer site. These survived ADSCs at the wound site could release more and longer biomolecules to optimize local immune environment [38, 39], as well as stimulating angiogenesis, thus resulting in an improvement of wound healing. In addition, the more survived ADSCs at the wound lesion may serve as a cell source that can directly differentiate into endothelial cells or fibrocytes, thus enhancing the formation of granulation tissues at the skin wound. Regrettably, we still need more in-depth studies to prove our explanations.

This study has confirmed that H-ADSCs/ADM is a novel covering graft for treating diabetic wounds. However, there remain several limitations. Firstly, owing to the lack of the technology, it is hard to measure the exact oxygen concentration at the H-ADSCs/ADM-mediated DFU healing site. In this study, we selected 1% O_2_ oxygen tension as hypoxia culture parameter for constructing the H-ADSCs/ADM membrane. Logically speaking, this culture parameter may be inaccurate. Next step, we should seek a suitable technology to accurately measure the exact oxygen concentration at H-ADSCs/ADM-mediated DFU healing site and then used this oxygen concentration as a reference to optimize the hypoxia culture parameters. Secondly, our animal experiments were conducted in streptozotocin-induced diabetic rats, which cannot completely imitate the type 2 diabetes observed in the clinic. Thus, the H-ADSCs/ADM membrane of this study may not be simply translated to treat DFU of humans. In the next step, diabetic rats with type 2 diabetes should be used to test the function of our graft again. Thirdly, given the secretion of angiogenic and anti-inflammatory molecules, the implanted ADSCs with the potential of stimulating tumor growth cannot be ignored in clinical setting. Despite these limitations, this study provides a basis for the clinical application of the H-ADSCs/ADM membrane in the treatment of diabetic wound.

## 5. Conclusion

In summary, a novel tissue-engineered dressing membrane (H-ADSCs/ADM) was constructed by culturing ADSCs on an ADM membrane under a hypoxia condition. This study indicated that ADM is an ideal biomaterial for ADSC delivery in a wound healing scenario. Additionally, in vitro results showed that the ADSCs in the ADM cultured under a hypoxia condition secreted more VEGF-A, and their culture medium presented better properties in promoting the proliferation, migration, and tube formation of HUVECs. In vivo results showed hypoxia-culture mode could prolong the cell survival time of H-ADSCs/ADM membrane at the chronic ulcer site, consequently improve angiogenesis, inhibit inflammation, and increase extracellular matrix remodeling, eventually accelerating DFU closure. This study showed that H-ADSCs/ADM membrane may offer innovative strategies for DFU treatment in the clinic.

## Figures and Tables

**Table 1:** Primer sequences used for qRT-PCR analysis.

Gene	Primer sequence	Species
IL-1*β*	Forward: 5′-ATAGCAGCTTTCGACAGTGAG-3′ Reverse: 5′-GTCAACTATGTCCCGACCATT-3′	Rat
IL-6	Forward: 5′-GACTGATGTTGTTGACAGCCACTGC-3′ Reverse: 5′-TAGCCACTCCTTCTGTGACTCTAACT-3′	Rat
NF-*κ*B	Forward: 5′-CGATCTGTTTCCCCTCATCT-3′ Reverse: 5′-TGCTTCTCTCCCCAGGAATA-3′	Rat
*β*-Actin	Forward: 5′-ATCTGGCACCACACCTTC-3′ Reverse: 5′-AGCCAGGTCCAGACGCA-3′	Rat

## Data Availability

The raw data supporting the conclusions of this article will be made available by the authors, without under reservation.
